# Rheumatoid Arthritis and Associated Lung Diseases: A Comprehensive Review

**DOI:** 10.7759/cureus.22367

**Published:** 2022-02-18

**Authors:** Ahmad T Azam, Oladipo Odeyinka, Rasha Alhashimi, Sankeerth Thoota, Tejaswini Ashok, Vishnu Palyam, Ibrahim Sange

**Affiliations:** 1 Internal Medicine, Allama Iqbal Medical College, Lahore, PAK; 2 Internal Medicine, University of Ibadan College of Medicine, Ibadan, NGA; 3 Internal Medicine, University of Baghdad College of Medicine, Baghdad, IRQ; 4 Internal Medicine, Meenakshi Medical College Hospital and Research Institute, Kancheepuram, IND; 5 Internal Medicine, Jagadguru Sri Shivarathreeshwara (JSS) Medical College, Mysore, IND; 6 Internal Medicine, Jagadguru Jayadeva Murugarajendra (JJM) Medical College, Davanagere, IND; 7 Research, Karamshi Jethabhai (KJ) Somaiya Medical College, Hospital and Research Center, Mumbai, IND

**Keywords:** rheumatoid arthritis, rheumatoid disorder, interstitial lung disease, complication of rheumatoid arthritis, progressive interstitial lung disease

## Abstract

Rheumatoid arthritis (RA) is a prevalent autoimmune disorder affecting 0.5-1% of the population in North America and Europe. Pulmonary manifestations in rheumatoid arthritis patients result in significant morbidity and mortality. Management of these pulmonary manifestations in RA patients causes various challenges for the physicians. This review article has discussed the current state of knowledge of these pulmonary manifestations, including interstitial lung diseases, airway-related diseases, pulmonary vasculature, and pleural involvement in RA patients. This review article has also explored various pharmacological options, including steroids, disease-modifying antirheumatic drugs (DMARDs), immunosuppressive drugs, and biologic agents. Non-pharmacological options include conservative treatment, supplemental oxygen, pulmonary rehabilitation, smoking cessation, and lung transplantation.

## Introduction and background

Rheumatoid arthritis (RA) is one of the most prevalent chronic inflammatory diseases characterized by progressive autoimmune and inflammatory damage to cartilage, bones and synovial lining of the joints eventually resulting in the severe disability of the patient [1,2]. The prevalence of RA in North America and Europe has been reported to be around 0.5-1% [1]. However, the prevalence and incidence of RA have been a recent topic of debate. Several studies reported that the incidence of rheumatoid arthritis decreased during the latter half of the 20th century. However, Myasoedova et al. reported that the incidence of RA has been on the rise again since 1995 [1,3]. According to a recent observational study by Hunter et al., the prevalence of RA has increased from 2004 to 2014 [4]. The female sex is predominantly more affected than their male counterpart [1]. Other major risk factors include old age, smoking, family history of the disease, and obesity [5]. RA can typically affect any age group but is more common among people aged 30-50 [5]. Pathophysiology of RA involves infiltration of T-lymphocytes, B-lymphocytes and monocytes in the synovial membrane of different joints of the body [6]. Pro-inflammatory cytokines including interleukin-1 (IL-1), tumor necrosis factor (TNF), and interleukin-6 (IL-6) released by macrophages mediate the damage to the surrounding bone and cartilage [7]. Activation of endothelial cells and proliferation of fibroblast-like cells in the synovial membrane result in neovascularization and pannus formation, respectively [8]. The etiology of rheumatoid arthritis is multifactorial [9]. Genetic susceptibility plays a major role and increases the risk of developing RA threefold to ninefold. HLA-DRB1 (Human Leukocyte Antigen DR Beta 1) region is strongly associated with anti-cyclic citrullinated peptide antibodies (ACPA) positive RA [10]. Several triggers, including smoking, infections and dust particles, have been studied for their role in causing RA [9]. However, smoking has been the most consistent risk factor associated with RA [11]. RA classically presents as a symmetrical polyarthritis primarily affecting small joints of hands and feet. The patients of RA mostly present with the complaint of pain and swelling in multiple joints of the body that is accompanied by morning stiffness that stays for more than 30 minutes but gets better with activity [12]. When left untreated, RA can cause joint deformities and atlantoaxial subluxation, which can eventually lead to severe disability of the patient [13]. Extra-articular manifestations (EAM) are common in the later stages of the disease and involve rheumatoid nodules near bony prominences, fever, weight loss, and myalgias [12]. Pulmonary manifestations include pleural effusions, interstitial lung diseases, and pleuritis [14]. Cardiovascular manifestations include pericarditis, myocarditis, coronary vasculitis, and enhanced risk of congestive heart failure [15]. Neurological manifestations include median nerve compression, mononeuritis multiplex, and entrapment neuropathy secondary to synovitis [16]. Other EAM includes osteoporosis, Felty's syndrome, glomerulonephritis, and scleritis [17]. Early diagnosis and treatment of RA have shown promising clinical outcomes for the patients, including a higher possibility of drug-free remission and decreased damage to the joints [18]. The diagnosis of RA is primarily clinical as there are no set diagnostic criteria established yet. However, 2010 ACR/EULAR (American College of Rheumatology/ European League Against Rheumatism) Classification criteria for RA can help the physicians establish the diagnosis [19]. X-ray, ultrasound, and magnetic resonance imaging (MRI) can assess the severity of damage to the joints [20]. Serologic testing for autoantibodies, including ACPA and rheumatoid factor (RF), can be helpful in the diagnosis but up to 30% of RA are negative for these autoantibodies [12]. Treatment should be initiated as early as possible after a diagnosis of RA has been made. The primary treatment goals involve decreasing pain and swelling in the joints, reducing radiological disease severity, improving the patient's quality of life, and controlling extra-articular manifestation of rheumatoid arthritis [21]. Disease-modifying antirheumatic drugs (DMARDs) are the most common treatment options for RA patients. Monotherapy with methotrexate and corticosteroids is recommended as the first line of treatment for RA patients [21]. Combination therapy with two or more DMARDs can be used in case of failure of monotherapy. Biologic DMARDs, including anti-TNF agents (adalimumab, golimumab) can be added to therapy if disease remission is not achieved with synthetic DMARDs. Nonsteroidal anti-inflammatory drugs (NSAIDs) and corticosteroids help decrease inflammation [21]. Pulmonary manifestations are one of the most challenging complications faced during the management of RA patients. RA-associated interstitial lung diseases (RA-ILD), pleural effusion, pleuritis, bronchiectasis, pulmonary vascular diseases, and drug-associated lung complications are major pulmonary manifestations caused by RA [22]. It is important to probe into the challenges faced by physicians and patients due to high morbidity and mortality due to pulmonary complications in RA. This review article aims to review the spectrum of pulmonary manifestations in RA, discuss the latest pharmacological and non-pharmacological options for managing RA-associated lung diseases and discuss the challenges physicians face in managing this morbidity.

## Review

Rheumatoid arthritis is associated with a wide range of pulmonary manifestations. Some of the major RA-associated lung diseases are discussed below:

RA-associated interstitial lung diseases

Interstitial lung disease (ILD) is one of the most prevalent and well-studied pulmonary manifestations of rheumatoid arthritis [23]. Most commonly diagnosed RA-ILDs include usual interstitial pneumonia (UIP) and non-specific interstitial pneumonia (NSIP) [24]. However, diffuse alveolar damage, organizing pneumonia (OP), acute interstitial pneumonia, and obliterative bronchiolitis have also been reported in RA patients.

**Figure 1:**
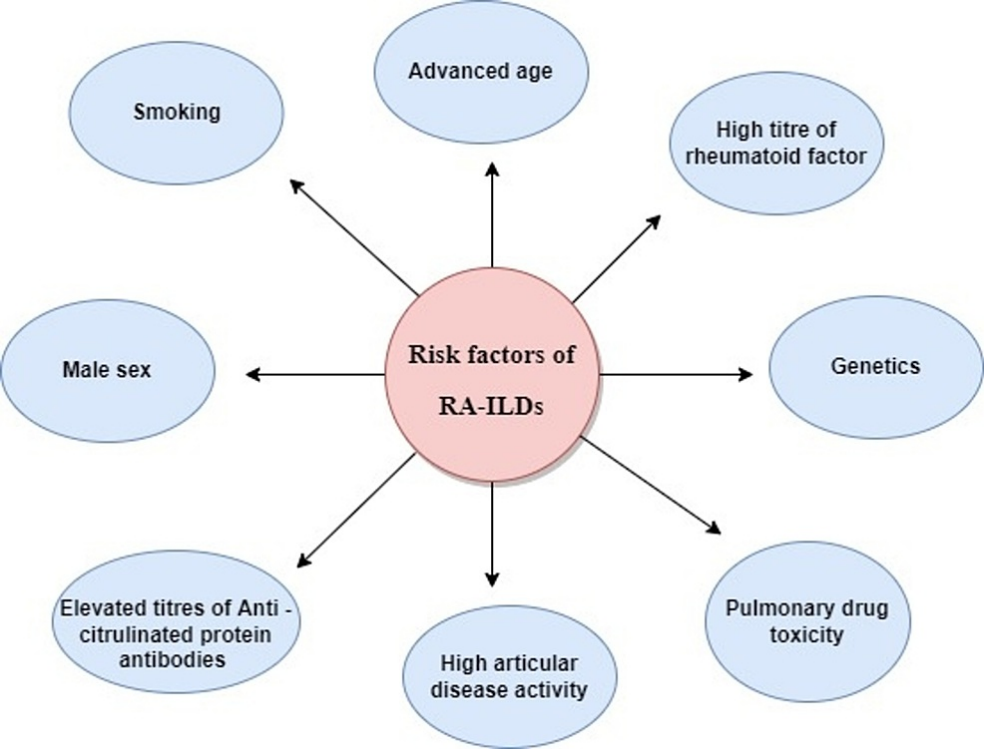
Risk factors of RA-ILD RA - Rheumatoid Arthritis, ILD- Interstitial Lung Diseases

The pathogenesis of RA-ILD is still not well understood; however, genetics and environmental factors play a significant role in the manifestation of ILD in RA patients [14]. Smoking, advanced age, male sex, high-titer anti-cyclic citrullinated peptide antibodies, high-titer rheumatoid factor, and genetics are major risk factors of RA-ILDs (Figure 1).

HLA-DRB1, HLA-DR4, and HLA-B40 are some of the significant HLA variants that play their role in the manifestation of ILDs in RA patients [25]. Smoking has been found to play an important role in triggering the immune reaction to citrulline-modified proteins by interacting with HLA-DR shared epitope (SE) genes [26]. A population-based case-control study was conducted in Sweden in 2006 that included 1544 participants (913 cases and 631 controls). It was found in this study that the previous history of smoking and the presence of two copies of HLA-DR SE genes enhanced the risk for rheumatoid arthritis by 21-fold as compared to non-smokers with no SE genes [26]. Several epidemiological studies have also reported an undeniable association between smoking and RA-ILDs [27]. Lung parenchymal and airway damage due to environmental exposure results in enhanced citrullination of proteins [28]. This initiates an inflammatory process and activation of cytokines, chemokines and growth factors, such as tumor necrosis factor (TNF), interleukins (IL), and vascular endothelial growth factor (VEGF) in a genetically predisposed individual [29]. Proliferation and differentiation of fibroblasts cause matrix metalloproteinases (MMP) hyperactivity and enhanced deposition of extracellular matrix (ECM); this results in the development of ILD and pulmonary fibrosis [27]. However, Zhang et al. recently reported that Interleukin-17 (IL-17) can have a major role in the pathogenesis of RA-ILD as lung biopsies of RA-ILD patients had demonstrated higher IL-17 expression in areas of fibrosis [30].

Clinical features of RA-ILDs include cough of insidious onset, external dyspnea, fatigue, and generalized weakness. Symptoms generally progress slowly, but the rate of progression varies from one patient to another [27]. It also varies among different histological subtypes [24]. A study was conducted by Kim et al. at the University of California, San Francisco and the Mayo Clinic, United States of America (USA) between 2001 and 2008. The study included a sample population of 82 patients with RA-ILD, which was followed for a median duration of 3.9 yrs (range 0.3-7.5 yrs). RA-UIP cohort had a median survival time of 3.2 years which was worse than the non-UIP cohort, which had a median survival time of 6.6 years. It concluded that UIP has a faster rate of progression and worse survival rate than other histological subtypes of ILD in RA patients [31].

Imaging plays a critical role in the diagnosis of RA-ILDs. Chest radiograph and high-resolution computed tomography (HRCT) are the initial investigations to identify the distribution and patterns of interstitial parenchymal abnormalities. HRCT has significantly higher diagnostic sensitivity as compared to chest radiographs. A study was conducted by Dawson et al. in a sample population of 150 patients with RA; HRCT evidence of ILD was observed in 28 patients (19%). On the other hand, findings consistent with ILD were observed in chest radiographs in four patients (<3%). This concluded that chest radiograph is not independently predictive investigation in patients with high suspicion of RA-ILD and abnormal chest radiographic findings warrant the need for HRCT to rule out RA-ILD in such patients [32].

The most common radiological feature in the UIP is subpleural and basilar predominant reticular abnormalities [33]. Honeycombing and traction bronchiectasis are also present, but ground-glass opacification is usually absent [33]. On the other hand, NSIP is characterized by ground-glass opacification with minimal honeycombing or architectural distortion [34]. Pulmonary function tests (PFTs) can be helpful diagnostic modality especially in the absence of symptoms. In patients with RA-ILD, PFTs may show a pattern of restrictive lung disease with a reduced diffusing capacity of the lung for carbon monoxide (DLCO) [35]. Bronchoalveolar lavage (BAL) in patients with RA-ILD yields non-specific results. Lymphocytosis is the most common finding in most patterns except in UIP. Neutrophilia is more typical in patients with UIP patterns [36]. Bronchoalveolar lavage and surgical lung biopsy can be helpful diagnostic modalities if the diagnosis is uncertain or findings on HRCT are atypical [27].

Early detection of RA-ILD results in a significant decline in disease progression [37]. A study was conducted by Gochuico et al. involving a sample population of 64 adults with RA and 10 adults with rheumatoid arthritis-related pulmonary fibrosis (RAPF). Twenty-one of 64 (33%) RA patients without pulmonary symptoms had preclinical ILD detectable by HRCT. Follow-up of this sample population mean follow-up time period being 2.5 years, reported that radiological disease detectable on HRCT progressed in 12 of 21 subjects (57%). This study concluded that asymptomatic pulmonary manifestations, including ILDs that are only detectable by HRCT, can be progressive in patients with RA [37].

Given the significant prevalence of pulmonary manifestations in RA, physicians should have a low threshold to proceed with a detailed evaluation of pulmonary symptoms in RA patients [38]. The prognosis varies with different histological subtypes, and studies have demonstrated that the UIP subtype exhibits the worst prognosis of all the RA-ILDs [39]. A study was conducted by Olson et al. to ascertain mortality rates from RA-ILDs in the United States from 1988 through 2004, demonstrating that overall mortality from RA is declining; however, mortality from RA-ILD is on the rise, especially in older age groups [40]. In another cohort study by Bongartz et al., which included a sample population of 582 patients with RA and 603 control subjects without RA, it was indicated that there was a 13% increased risk of mortality due to ILD in RA patients as compared to the general population. This study also observed that the risk of mortality for RA patients with ILD was threefold higher than in RA patients without ILD [41].

RA-related airway diseases

Most commonly seen airway-related manifestations in RA patients include bronchiectasis, bronchiolitis, and cricoarytenoid arthritis [42]. It has been observed that 39 to 60% of RA patients develop airway-related morbidities in their lifetime [43]. HRCT and PFTs can be very helpful in diagnosing large airway involvement, although HRCT has a tremendous diagnostic sensitivity for small airway involvement in RA patients [42].

Large airway abnormalities include bronchiectasis and cricoarytenoid arthritis. Bronchiectasis has been reported in around 30 percent of RA patients [27]. It typically presents with insidious cough and productive sputum [44]. Various theories have been proposed regarding the pathogenesis of bronchiectasis in RA patients. Chronic suppurative infections and the use of DMARDs in the treatment of RA are the leading causes of bronchiectasis in RA patients. A longitudinal cohort study was conducted by Geri et al. between 2000 and 2009 that included 47 RA patients with bronchiectasis. After a mean follow-up per patient of 4.3 ± 3.1 years, it was observed that the use of biological agents in the treatment of RA and sputum colonization by bacterial organisms are independent risk factors for lower respiratory tract infection [45]. Cricoarytenoid joint arthritis presents with hoarseness, throat pain, dyspnea, and stridor due to midline adduction of the vocal cords. It primarily occurs due to synovial thickening of the cricoarytenoid joint, resulting in continuous cartilage erosion. HRCT of the neck can diagnose the condition before the appearance of clinical symptoms. Complications include acute stridor or obstructive respiratory failure [46].

Small airway abnormalities due to RA include follicular and constrictive bronchiolitis [22]. Follicular bronchiolitis may present with exertional dyspnea, nonproductive cough, and wheezing. HRCT is the mainstay of diagnosis and can demonstrate findings before clinical symptoms. Follicular bronchiolitis is characterized by hyperplasia of bronchial-associated lymphoid tissue. Presence of centrilobular peribronchial nodules less than 3mm in size is detectable on HRCT [47]. Unlike other rheumatoid pulmonary manifestations, obliterative bronchiolitis usually has a more severe and acute clinical presentation. It may present with cough, bronchorrhea, and rapidly progressive dyspnea without systemic symptoms. Constrictive bronchiolitis is characterized by damaged airway epithelium resulting in airflow obstruction. HRCT findings include mosaic attenuation, bronchial wall thickening, and centrilobular nodules [48].

RA-associated pleural diseases

Pleuritis and pleural effusion are the most prevalent pleural manifestations observed in RA patients. The pleural disease in RA patients can occur before or alongside the first clinical signs of joint involvement. Pleural disease is primarily silent in RA patients with symptomatic disease present in only 3-5% of the patients [49]. Large pleural effusions can present with cough, dyspnea, chest pain, and fever [50].

Pathogenesis is still not well understood. It has been proposed that IgG, IgE, and IgM RF antibodies are produced by plural mononuclear cells which contribute to the formation of immune complexes [51]. These immune complexes damage the capillary endothelium and increase the capillary permeability of pleural space. Other proposed mechanisms include high protein content in rheumatoid pleural effusion and blockage of lymphatic drainage in inflamed parietal pleura contributes towards the formation of rheumatoid pleural effusion [51].

Ultrasound-guided thoracocentesis is usually considered first-line investigation in patients with high suspicion of the disease. Pleural fluid in RA usually has low pH (< 7.3) and glucose levels (< 50 mg/dL) [51]. Rheumatoid factor is usually a characteristic finding in the pleural fluid analysis of RA patients but its absence doesn't rule out the possibility of the disease. Cytological analysis of pleural effusion reveals multinucleated giant cells, macrophage predominance, and granulomatous debris. RA pleural effusions contain a high titer of RF, raised adenosine deaminase levels, decreased Complement total (CH50) levels [50]. Medical thoracoscopy and pleural biopsy are required if the diagnosis is still unclear on thoracocentesis. It has been reported that severe or untreated pleural effusion can eventually result in fibrothorax and lung restriction [49]. The risk of empyema in RA patients is still not well studied; however, it has been hypothesized that patients treated with immunosuppressive therapy had a higher risk of developing empyema [52]. However, clinical trials with a large sample population are not available to support this hypothesis.

Other RA-associated pulmonary manifestations

Other uncommon RA-associated pulmonary manifestations include rheumatoid pulmonary nodules, rheumatoid pulmonary vasculitis, and drug-induced pulmonary complications [53]. Rheumatoid pulmonary nodules can occur in RA patients when exposed to inorganic dust, including asbestos, silica, and coal. A prospective study by Zrour et al. with a sample population of 75 RA patients undergoing HRCT reported rheumatoid pulmonary nodules in 4% of patients [54]. The disease was clinically silent in 4% of these cases. HRCT is the modality of choice to detect rheumatoid nodules as they can be missed in the regular chest radiographs [54]. There is no recent data on the incidence of this manifestation in RA patients [54]. Rheumatoid pulmonary vasculitis can most commonly manifest as pulmonary angiitis in 1% of RA patients [55]. It presents along with cutaneous vasculitis and vasculitis neuropathy [55]. Drug-induced pulmonary complications of different drugs used by RA patients are discussed in the management section below.

Updated management guidelines

Early treatment should be started in patients showing clinical or radiologic manifestations of the disease. The disease severity and rate of progression of the disease in RA-ILD patients can be used to identify the need to start or augment the therapy to manage pulmonary manifestations of RA [27]. Treatment includes supportive measures and the use of anti-inflammatory agents to reduce the inflammation involved in the pathogenesis of the disease.

Conservative Management

Conservative management of RA-ILD patients is usually considered in the case of mild disease or patients with multiple comorbidities and advanced age due to contraindication of pharmacological management [27]. Due to the well-documented role of smoking in the pathogenesis of RA-ILD, it is important to provide smoking cessation counseling to the patients and educate them regarding its implications in the worsening of the disease [27]. Supplemental oxygen has also proven to be an important part of a palliative protocol for patients with severe disease [56]. Several studies have shown that supplemental oxygen improves endurance time, walking distance, and dyspnea in ILD patients [56]. However, there are no clinical trials available with a sufficient patient population to support this evidence. Pulmonary rehabilitation has shown benefits in improving pulmonary symptoms in patients with idiopathic pulmonary fibrosis (IPF), so it can be theorized that it will help RA-ILD patients as well. However, functional limitations of RA patients restrict their ability to perform exercises involved in this intervention [57]. This warrants the need for tailored pulmonary rehabilitation protocol for RA-ILD patients. Psychosocial support, Pneumococcal, and Influenza vaccination are other conservative interventions recommended for RA-ILD patients [58].

Pharmacological Management

Several treatment options are recommended based on updated clinical studies. Below, we discuss the rationale behind each recommendation for pharmacological management of RA-ILD patients.

Immunosuppressive Drugs

Glucocorticoids are generally considered the first line of management for patients with RA-ILDs. It is recommended to start with oral Prednisone at a daily dose of 0.5 mg/kg with subsequent tapering based on the clinical response of the patient [59]. In a retrospective study by Song et al. on 84 patients with RA-related UIP, 50% of the patients showed improvement in their clinical symptoms when treated with glucocorticoids alone or in combination with DMARDs [60].

It is suggested that the survival rate of patients with ILD increases with the use of another immunosuppressive drug, Azathioprine. A prospective, double-blind, randomized, placebo-controlled study was done by Raghu et al. involving 27 newly diagnosed patients with IPF. Fourteen patients were treated with Azathioprine and Prednisone and 13 with Prednisone alone. The study reported an improved survival rate in a cohort treated with a combined regimen of steroids and Azathioprine [61]. However, a recent trial, “Prednisone, Azathioprine, N-acetylcysteine: A Study That Evaluates Response in Idiopathic Pulmonary Fibrosis (PANTHER-IPF study)” reported that mortality is increased in the patients treated with Azathioprine and Prednisone. This study had to be discontinued prematurely due to the high mortality rate in patients treated with Azathioprine and Prednisone [62].

Mycophenolate mofetil (MMF) is another potentially helpful immunosuppressive drug with encouraging results in several retrospective studies. MMF inhibits inosine monophosphate dehydrogenase (IMPDH), eventually hindering de novo purine biosynthesis. It diminishes B and T lymphocyte proliferation, reducing inflammation [63]. In a study of 125 patients with connective tissue disease-related ILD treated with MMF by Fischer et al. in 2013, an analysis of 18 patients with RA-ILD showed that patients treated with MMF for a median of 897 days had improved forced vital capacity (FVC). Less than 10% of the patients in the study discontinued the treatment due to adverse effects of the drug [64]. In a meta-analysis by Tzouvelekis et al. with a sample population of 69 patients with systemic sclerosis-associated interstitial lung disease, it was reported that the use of MMF has improved disease stabilization and prevented forced vital capacity (FVC) decline [65].

DMARDs

A significant hurdle in the treatment of RA-ILD is the fact that many potential pharmacological options like DMARDs and biologic agents have proven record to cause pulmonary toxicity. Methotrexate (MTX) and anti-TNF agents should be used with caution in patients with RA-ILD [66]. Non-biologic DMARDs, including methotrexate and Leflunomide (LEF), have well-demonstrated ILD promoting effects, due to which they are not considered a primary therapeutic option in RA-ILD patients. A meta-analysis by Conway et al. encompassing 22 studies with 8,584 participants reported that MTX is associated with an increased risk of pneumonitis and other respiratory infections [66]. It is recommended to get pulmonary functions tests done before initiating MTX as most patients develop pulmonary complications within the first months of initiation of treatment [67].

Biologics

Anti-TNF agents have both antifibrotic and profibrotic properties that make their use controversial in the treatment of RA-ILDs [68]. Use of adalimumab, golimumab, infliximab, and etanercept has been linked with new-onset or exacerbation of ILDs [69-72]. In one study by Perez-Alvarez et al., 122 reported cases of new-onset or worsened ILD in the setting of anti-TNF use were evaluated. A total of 108 cases used in the study were of RA patients. 97% of cases of ILD reported in the study were linked with the use of anti-TNF agents including etanercept and infliximab [73]. In contrast, a prospective observational study by Dixon et al. involving a sample population of 367 patients with RA-ILD demonstrated that the mortality rate was not increased after treatment with anti-TNF agents compared with traditional immunomodulatory drugs (Table 1) [74]. Several cases have been reported in which stabilization of pulmonary function was seen in patients using anti-TNF agents [75]. However, the evidence available regarding the efficacy of anti-TNF agents in RA-ILD patients is insufficient.

**Table 1:** Summary of studies demonstrating pathophysiology and management of rheumatoid arthritis-associated interstitial lung diseases RA - Rheumatoid Arthritis, DMARDS - Disease-Modifying Antirheumatic Drugs, ILD - Interstitial Lung Diseases, UIP - Usual Interstitial Pneumonia, TNF - Tumor Necrosis Factor, HRCT - High-Resolution Computed Tomography, HLA - Human Leukocyte Antigen, SE - Shared Epitope

References	Design	Number of participants	Methods	Conclusion
Klareskog et al. (2006) [26]	Population-based case-control study	1544 (913 cases + 631 controls)	Cases were individuals aged 18–70 years with newly diagnosed RA. There was a random selection of controls from the Swedish National Population Registry.	Previous history of smoking and the presence of two copies of HLA–DR SE genes enhanced the risk for rheumatoid arthritis by 21-fold.
Kim et al. (2010) [31]	Cohort study	82	82 patients with RA-ILD (identified retrospectively) participated in the study between 2001 and 2008.	UIP has a faster rate of progression than other histological subtypes of ILD in RA patients.
Dawson et al. (2001) [32]	Cohort study	150	150 patients with RA	HRCT evidence of ILD was observed in 19% of patients but evidence of ILD on chest radiograph was seen in 3%.
Gochuico et al. (2008) [37]	Cohort study	74	64 adults with RA and 10 adults with rheumatoid arthritis-related pulmonary fibrosis.	33% of RA patients without pulmonary symptoms had preclinical ILD detectable by HRCT.
Bongartz et al. (2010) [41]	Cohort study	1185 (582 cases and 603 control)	582 RA patients 603 control subjects were followed for a mean of 16.4 and 19.3 years, respectively.	Risk of mortality for RA patients with ILD was threefold higher than in RA patients without ILD.
Song et al. (2103) [60]	Retrospective study	84	84 patients with RA-UIP	50% of the patients showed improvement in their clinical symptoms when treated with glucocorticoid alone or in combination with DMARDs.
Dixon et al. (2010) [74]	Prospective observational study	367	367 patients with RA-ILD	Mortality was not increased after treatment with anti-TNF agents compared with traditional DMARDs.

Rituximab is a monoclonal antibody against CD20 B-cell marker and is currently being studied as a potential treatment for RA-ILD patients. Several studies have hypothesized that there can be a role of B-cells in the pathogenesis of RA-ILD. Atkins et al. reported significant follicular B cell hyperplasia in samples of open lung biopsies of RA-ILD patients [76]. Small observational studies and case reports have shown promising results regarding the efficacy of rituximab, but they are still inconclusive. Becerra et al. did a cohort study on 264 RA patients being treated with rituximab; 38 of them had lung involvement before initiation of treatment. No evidence of progression of lung symptoms was found in RA-ILD patients after a median follow-up period of 2.5 years, but no improvement was also seen in their pulmonary symptoms [77]. Clinical trials with a larger cohort of patients are required to delineate the safety and efficacy of rituximab in this subset of the population.

Surgical intervention

Lung Transplant

A lung transplant can be considered for young patients with advanced refractory disease. However, it is contraindicated in patients with advanced age, multiple comorbidities, immobility, and other extra-articular manifestations. A systematic review by Richardson and Singer reported a 5-year survival rate from 46% to 76% in systemic sclerosis ILD patients who received a lung transplant [78].

Future implications

New treatment options are being studied, including pirfenidone, an antifibrotic agent. A study by Wu et al. has demonstrated antifibrotic effects of pirfenidone in RA-ILD lung specimens. A transition of fibroblast to myofibroblast was inhibited by down-regulation of Smad3-ATF3 signaling [79]. Genome-wide association studies are being conducted on RA-ILD patients, which will provide a deeper insight into the pathogenesis of the disease [58]. Researchers are trying to find accurate and clinically practical biomarkers which can enable clinicians to make an early diagnosis of the disease, eventually resulting in early initiation of treatment [80].

Limitations

Our literature review does not provide a standard treatment algorithm that can be followed for RA-ILD patients due to the lack of randomized controlled trials (RCTs) in this subset of the population. Treatment protocols need to be individualized for each patient. This study does not address the growing importance of unique biomarkers that physicians can use for an early diagnosis of RA-ILD patients.

## Conclusions

This review article discussed the spectrum of pulmonary manifestations in RA patients, updated management guidelines, and challenges faced by the physicians during the management of such patients. The clinical implication of this review article is to summarize the current state of knowledge of pulmonary manifestations in RA patients which will help the physicians better understand the disease and novel treatment protocols for RA-ILD patients. We discussed the challenges faced by the clinicians while managing such patients and how they can be tackled through a tailored management approach for each patient. Major pulmonary manifestations include ILDs, airway-related diseases, and pleural diseases, including pleuritis and pleural effusion. Pathogenesis of RA-ILD has been associated with genetics and environmental exposures, including smoking. Chest radiograph, HRCT, and PFTs are considered major diagnostic modalities. Treatment is tailored for each patient depending on various factors and is begun by non-pharmacological measures such as smoking cessation, supplemental oxygen therapy, and chest physiotherapy. As per the studies reviewed above, steroids are generally the first line of treatment. Other immunosuppressive drugs and biologic agents can be added to the regimen after carefully considering the patient's previous medical history. Lastly, we recommend more future studies, primarily controlled therapeutic trials with a sufficient patient population to identify novel and safe therapeutic options in this patient population.
